# Combination therapy with bone marrow-derived mesenchymal stem cell transplantation and propolis improves streptozotocin-induced kidney injury in diabetic rats

**DOI:** 10.22038/ajp.2025.26229

**Published:** 2026

**Authors:** Fatemeh Salami, Sara Hosseinian1, Elahe Mahdipour, Samira Shahraki, Mohammad Hossein Rigi, Zahra Samadi Noshahr, Hossein Hosseinzadeh, Abolfaz l Khajavi Rad

**Affiliations:** 1 *Department of Physiology, Faculty of Medicine, Mashhad University of Medical Sciences, Mashhad, Iran*; 2 *Applied biomedical Research Center, Basic Sciences Research Institute, Mashhad University of Medical Sciences, Mashhad, Iran*; 3 *Department of Medical Biotechnology and Nanotechnology, Faculty of Medicine, Mashhad University of Medical Sciences, Mashhad, Iran*; 4 *Department of Physiology, School of Medicine, Zahedan University of Medical Sciences, Zahedan, Iran *; 5 *Department of Physiology, Faculty of Nurcing and Midwifery, Chabahar University of Medical Science *; 6 *Department of Pharmacodynamics and Toxicology, School of Pharmacy, Mashhad University of Medical Sciences, Mashhad, Iran*; 7 *Pharmaceutical Research Center, Pharmaceutical Technology Institute, Mashhad University of Medical Sciences, Mashhad, Iran*

**Keywords:** Diabetic nephropathy, Propolis, Mesenchymal stem cell, Honey

## Abstract

**Objective:**

The aim of the current study was to determine the therapeutic effects of combination therapy with propolis extract and rat bone marrow mesenchymal stem cells (MSCs) in streptozotocin (STZ)-induced diabetic rats.

**Materials and methods:**

Firstly, characterization of MSCs was performed and MTT assay was done to determine the optimum concentration of propolis for incubation with MSCs. Rats were divided into 8 groups: Control, diabetic , diabetic+propolis, diabetic+metformin, diabetic+MSCs, diabetic+MSCs+ propolis, diabetic+ MSCs pre-incubated with propolis. MSCs were transplanted via the tail vein on the 7^th^ and 21^st ^days of the study. Renal function tests and histopathologic examination were performed for all groups.

**Results:**

Serum glucose concentration in all propolis and MSCs treatment groups was significantly lower than that of the STZ group on the 21^st^ and 42^nd^ days of the study. On the 42^nd^ day, the concentration of serum albumin in the STZ group was significantly lower than the control. Serum albumin concentration in all diabetic groups treated with propolis and MSCs was significantly higher than the diabetic animals. On the 42^nd^ day, the concentrations of creatinine and urea in the STZ group were significantly higher than all the treatment and control groups. The renal index and histopathological parameters improved in all treatment groups compared with the STZ group.

**Conclusion:**

Our findings demonstrated, MSCs, propolis, and their combination demonstrate positive effects on renal function, kidney index, and histopathology in all treated animals compared with the STZ diabetic rats. These beneficial effects are comparable to those of metformin.

## Introduction

Diabetic kidney disease (DKD) is the main cause of end-stage renal disease (ESRD) worldwide. About 40% of newly diagnosed diabetic patients in Asian and Western countries have DKD (Alicic et al. 2017). The key risk factors for the development of DKD and induction of kidney injury are hypertension and hyperglycemia (Keane et al. 2003).

Propolis is a resinous mixture of different substances that honey bees gather from plant exudates and flowers (Sobocanec et al. 2006). It has antihyperglycemic properties in diabetic animals (El-Sayed et al. 2009). 

Propolis can also reduce proteinuria and kidney failure in diabetic animals (Noori et al. 2012). 

Mesenchymal stem cells (MSCs) therapy in DKD has recently attracted great research attention due to their clinically beneficial properties as a treatment modality, such as paracrine effects through secretory cytokines (Bruno et al. 2009). 

In addition, natural extracts can promote stem cell proliferation, differentiation and migration (Elkhenany et al. 2019). The current study aimed to determine the therapeutic effects of combination therapy with aqueous propolis extract and bone marrow MSCs on kidney function and histology in streptozotocin (STZ)-induced diabetic rats.

## Materials and Methods

### Chemicals

Streptozotocin (STZ) was purchased from Sigma-Aldrich Company (USA). Metformin was obtained from Razak Company (Tehran, Iran). Alpha-Minimum Essential Medium (α-MEM) and OsteoPlus and AdipoPlus differentiation mediums were purchased from Bio Idea (Tehran, Iran). Fetal bovine serum, trypsin, and penicillin-streptomycin were purchased from Biowest (France). 3-(4,5-dimethylthiazol-2-yl)-2,5-diphenyltetrazolium bromide (MTT) was purchased from Invitrogen (Germany). Rat CD44, CD90, CD34, and CD45 antibodies were purchased from Bio-Techne (USA). The aqueous propolis extract was purchased in the form of a commercially available 30% solution (Soren Tech Toos Company, Mashhad, Iran). 

### Animals

Forty-eight male Wistar rats weighing 250-300 g were obtained from the animal house of the Faculty of Medicine, Mashhad University of Medical Sciences, Mashhad, Iran. The animals were kept in standard rodent cages with 12 hr light/dark cycle and *ad libitum *access to food and water. All experimental procedures were approved by the Ethics Committee on Animal Research of Mashhad University of Medical Sciences (IR.MUMS.REC.1400.224).

### Study design and sample collection 

Forty-eight rats were randomly divided into eight groups (six rats in each group). For induction of diabetes, the rats were fasted for 6 hr before receiving a single dose injection of STZ (65 mg/kg, ip) dissolved in normal saline. Three days after STZ injection, rats with FBS (Fasting blood sugar) levels≥250 mg/dl were selected as diabetic. Treatment for all groups lasted six weeks and distilled water was used as a solvent for all orally administered medications. Experimental groups were described as follows: 

Control: injection of normal saline (instead of STZ) and normal saline daily by gavage for 6 weeks.

Diabetic: single dose injection of STZ (65 mg/kg, ip) and normal saline daily by gavage for 6 weeks (Furman 2015; Noshahr et al. 2020).

Diabetic + propolis extracts (200 and 400 mg/kg): diabetic rats treated with propolis (200 and 400 mg/kg, daily by gavage) for 6 weeks (Sameni et al. 2016).

Diabetic + metformin: diabetic rats treated with metformin (300 mg/kg, daily by gavage) for 6 weeks (Murai et al. 2017).

Diabetic + MSCs: diabetic rats treated with MSCs via tail vein at a concentration of 1×10^6^ in 0.2 ml PBS (Phosphate buffered saline) on the 7^th^ and 21^st^ days of the study (Paulini et al. 2016). 

Diabetic +MSCs+ propolis extract (200 mg/kg): diabetic rats treated with MSCs and propolis (200 mg/kg, daily by gavage) similar to the protocol mentioned above. 

Diabetic + pre-incubated MSCs: diabetic rats treated with pre-incubated MSCs (with 20% propolis for 48 hr) via tail vein at a concentration of 1×10^6^ in 0.2 ml PBS on the 7^th^ and 21^st^ days of the study. 

On the 1^st^, 21^st,^ and 42^nd^ days of the experiment, urine samples were collected using metabolic cages, and blood samples were taken from the orbital sinus (Sørensen et al. 2008). At the end of the study (day 42), blood was taken from the heart and both kidneys were collected and weighed. The blood samples were centrifuged at 3000 rpm for 15 min and then the serum was separated. 

### Isolation of MSCs

In this study, 4-week-old male Wistar rats were sacrificed with cervical dislocation and their femurs and tibiae were dissected, then, the skin and muscles were removed. Rat bones were placed in a 10-cm dish filled with α-MEM and one ends of the bones were chopped with a pair of scissors. The bone marrow was then aspirated through a 22G needle into a tube with α-MEM. Next, the cells were centrifuged at 600 g for 5 min, and the supernatant was collected. Cells were plated into a 25 cm^3^ cell culture flask in α-MEM containing 10% fetal bovine serum. Cultures were kept in an incubator at 37°C and 5% CO_2_. The culture media were changed every 3 days (Smajilagić et al. 2013). 

### Flow cytometry analysis

To verify the expression of MSCs-specific surface markers, flow cytometry analysis was used. The cells in 3rd passage were trypsinized and collected in a 15 ml tube, the supernatant was aspirated and the cells were washed with PBS. Then, 100 µl of the cell suspension was aliquoted into five tubes (1. isotype control; 2. CD44; 3. CD90; 4. CD34; and 5. CD45). Afterward, antibodies were added into each tube as follows: MSCs-specific surface markers including CD44 (CD44-FITC, BioLegend), and CD90 (CD90-PerCP/CY5.5, BioLegend), and hematopoietic cell-specific markers including CD45 (CD45-FITC- BioLegend), and CD34 (CD34-PE, BD Biosciences) and for the control, the MSCs stained by isotype control including IgG1-FITC, IgG1-PE, and IgG1-PerCP/CY5.5; and then all tubes were incubated at 4°C for 30 min. Afterward, cells were washed at 1500 rpm for 5 min with 500 µl of PBS. Finally, 250 PBS solution was added to the cell sedimentation and assessed by flow cytometry method (flow cytometry FACS Calibur (Becton Dickinson)). Eventually, the results were analyzed by FLOWJO software (Camilleri et al. 2016). 

### BM (bone marrow)-MSCs osteogenic differentiation

To assess osteogenesis, the differentiation culture medium composed of low glucose - Dulbecco's Modified Eagle Medium (L-DMEM), 10% FBS, 100 nM dexamethasone, 10 mM β-glycerophosphate, and 250 mM L-ascorbic acid, was added to the induced cells at a density of 2×10^5^ cells/well and 70% confluency. The culture media was changed two times per week for up to 3 weeks. These cells were fixed with 4% paraformaldehyde for 30 min at room temperature and stained with 0.1% Alizarin Red for 30 min at room temperature until calcium deposits were visible (Meesuk et al. 2022).

### BM-MSCs adipogenic differentiation

To assess adipogenesis, the cultured cells seeded in a 6-well plate and the adipocyte-inducing medium containing L-DMEM, 10% FBS, 1.0 mmol/l dexamethasone, 0.5 mmol/l isobutyl-methylxanthine, and 10 mg/l insulin, was added to the induced cells at a density of 2×10^5^ cells/well and 70% confluency for 12 days. Finally, cells were stained at room temperature with 0.3% Oil Red O (Meesuk et al. 2022).

### Determination of total phenolic contents

For the determination of total phenol content, Folin-Ciocalteu reagent was used. A dilute solution of 30% propolis aqueous extract or gallic acid (standard phenolic compound) was mixed with the Folin-Ciocalteu reagent (2.5 ml, 1:10 diluted with distilled water) and aqueous Na_2_CO_3_ (2 ml, 5%). After 30 min, the phenolic contents were specified by colorimetry at 765 nm (Wojdyło et al. 2007). The total phenolic quantity was determined as mg of gallic acid equivalent using an equation obtained from the standard gallic acid calibration graph. The concentrations of gallic acid as standard were: 0.025, 0.05, 0.1, 0.2, and 0.4 (mg/ml).

### Treatment of animals with MSCs

At first, MSCs were detached from the cell culture flasks using trypsin/EDTA and counted. About 1×10^6^ cells were considered for injection into each animal in cell groups. After centrifugation, the cell pellet was washed with PBS, and this pellet was quickly taken to the animal house for injection. Then, 100 µl of MSCs solution was injected into the tail vein slowly.

### MTT test

MTT test was used to evaluate propolis toxicity and also its optimum dose and time for MSCs' treatment. To do this, MSCs were seeded at a density of 10×10^3^ cells/well in 96-well plates and treated with 5%, 10%, 20%, and 30% (v/v) of propolis (900 μl media+100 μl propolis for 10%). After 24, 48, and 72 hr of incubation, the 20 μl of MTT solution (5 mg/mL) was added to each well. After 4 hr incubation, the medium was removed and 100 μl DMSO was added to dissolve formazan crystals. Finally, plates were examined using an ELISA microplate reader at 570 and 680 nm wavelengths (Pawlak et al. 2002).

### Serum biochemical measurement

Serum concentrations of urea, creatinine, albumin and glucose were determined using commercial kits according to the manufacturer's instructions (Pars Azmoon Company, Tehran, Iran). 

### Histopathological examination

Kidneys were fixed in 10% formalin, then the right kidneys were dehydrated in graded alcohols and molded in paraffin. Then, 5 µm tissue slices were formed, and the slides were stained with the hematoxylin-eosin method to be used in microscopic examinations. Samples were scored using a scoring system between 0 and 4 as follows: grade 0, no tissue damage; grade 1, <25% tissue destruction; grade 2, <50%; grade3, <75%; and grade 4, ≥75% tissue destruction (Havakhah et al. 2014; Kojima et al. 2007). 

### Kidney index 

The renal index was calculated based on the ratio of kidney weight to body weight for each animal.

### Statistical analysis

All data is expressed as means ± SEM. The difference between means was statistically analyzed using a one-way analysis of variance (ANOVA) followed by the Tukey test. Differences were considered statistically significant when p<0.05.

## Results

### Characterization of MSCs

#### Osteogenic and adipogenic differentiation of rat MSCs

**Figure 1:**
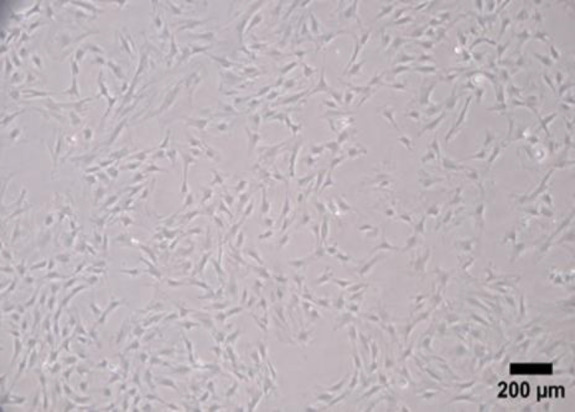
Microscopic image of rBM-MSCs (fusiform morphology and plastic-adherent capability) (passage number 3- 4X, scale bar: 200 µm).

The fusiform morphology and plastic-adherent ability of rat bone marrow mesenchymal stem cells (rBM-MSCs) are shown in Figure 1 (scale bar: 200 µm).

**Figure 2:**
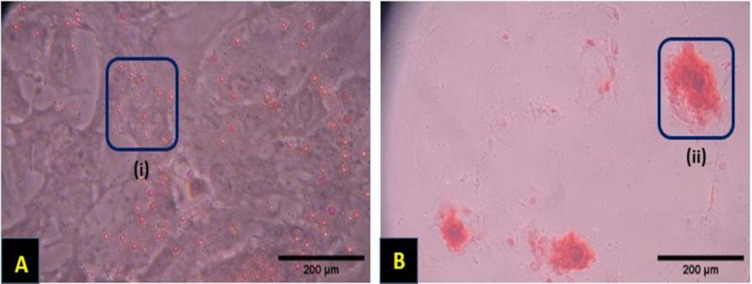
Differentiation potential of rBM-MSCs. (A) Adipogenic differentiation of rBM-MSCs. Lipid droplets were stained with Oil Red O. (B) Osteogenic differentiation of rBM-MSCs. Mineral deposits were stained with Alizarin Red. Squares (i and ii) indicate examples of positive staining.

 These cells were characterized as multipotent cells with the ability to differentiate into a variety of cell lineages including adipocytes and osteoblasts. Following oil red O and Alizarin red staining, the differentiated MSCs showed intracellular lipid droplets (Figure 2-A) and the production of calcium deposits (Figure 2-B).

### Flow cytometry

**Figure 3:**
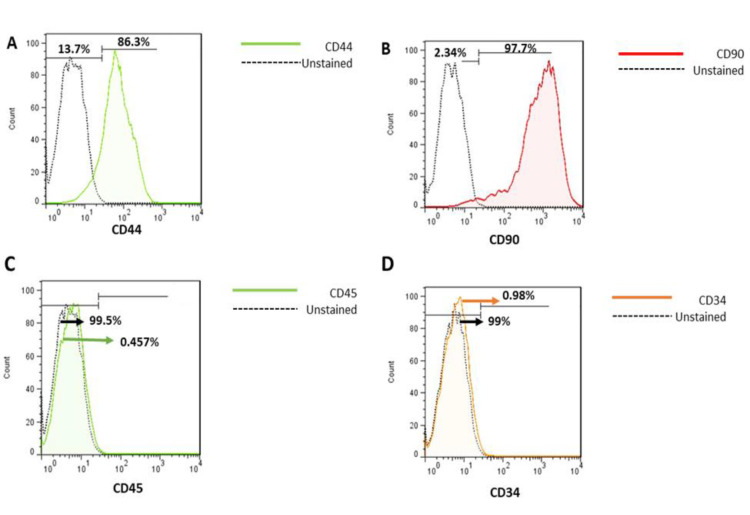
Flow cytometry analysis of rBM-MSCs. (A and B) Analysis of CD44 and CD90 as positive markers. (C and D) Analysis of CD45 and CD34 as negative markers.

We tested CD90 and CD44 expression to identify positive biomarkers of rat bone marrow mesenchymal stem cells and CD45 and CD34 as negative control. MSCs were stained with a fluorescent labeled mouse anti-rat CD90 (B) and CD44 (A) as positive antigens and negatively stained with monoclonal antibodies against CD34 (D), and CD45 (C) (Figure 3). Expression of CD90 and CD44 on MSCs was 97.7% CD90 and 86.3% CD44. CD marker expression may be affected by cell density, therefore the cells were stained at 80–100% confluency to eliminate this as a variable between experiments. Representative graphs related to each biomarker expression are illustrated in Figure 3.

### MTT assay

**Figure 4:**
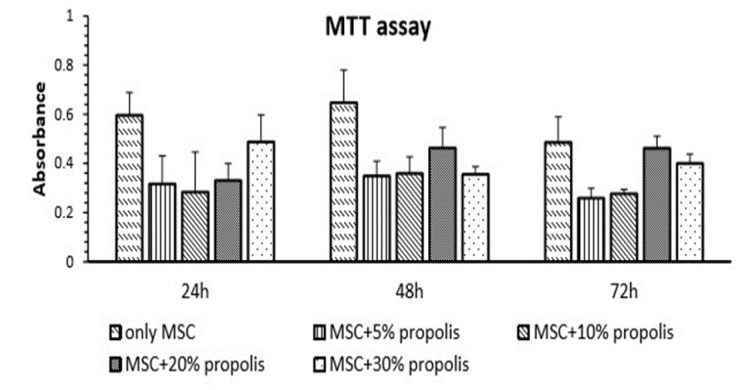
The results of MTT assay on MSC cells after treatment with different concentrations of propolis during 24, 48 and 72 hr. Values are mean±SEM (n = 6 per group) and were analyzed using one-way ANOVA followed by Tukey's post hoc test.

As shown in Figure 4, treatment with propolis at various concentrations over varying incubation periods of 24, 48, and 72 hr, did not significantly affect the metabolic activity of the cells when compared to the control group. However, the mean metabolic activity of cells treated with 20% propolis for 48 hr, was higher than other groups. Thereby, further experiments were proceeded by treating the cells with 20% propolis for 48 hr.

### The phenolic content of the propolis extract

 The phenolic quantity of propolis extract which was 4.7%. It was calculated as gallic acid equivalent.

### Serum biochemical parameters

#### Fasting blood sugar

**Figure 5:**
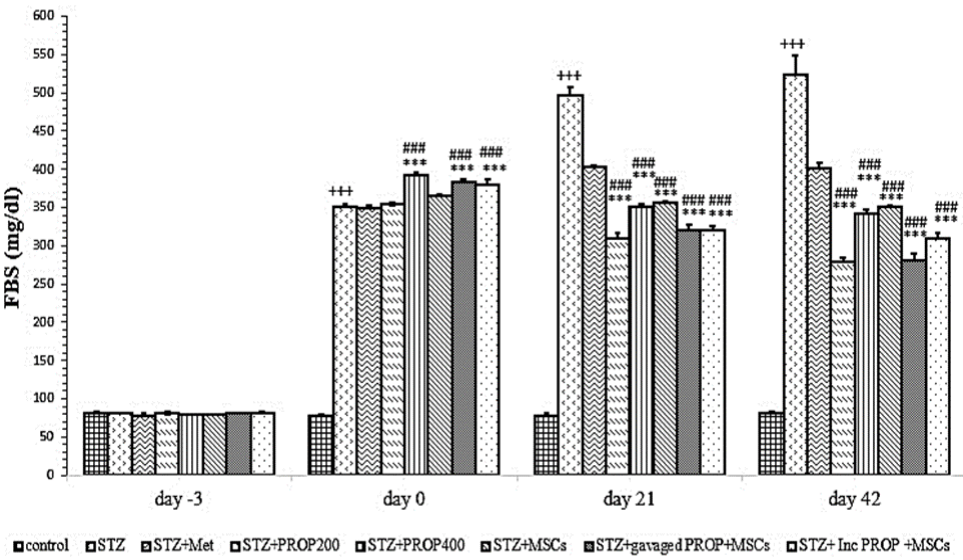
FBS in all experimental groups. Values are mean±SEM (n = 6 per group) and were analyzed using one-way ANOVA followed by Tukey's post hoc test. +++p<0.001 compared to control group, ***p<0.001 compared to STZ group, ^###^p<0.001 compared to STZ+Met group. STZ: Streptozotocin; Met: Metformin; PROP: Propolis; MSCs: Mesenchymal stem cell, Inc: Incubated.

As shown in Figure 5, FBS before STZ injection (day -3) showed no significant change between different experimental groups. However, on days 0, 21 and 42, FBS increased significantly in the diabetic group compared to the control group (p<0.001 for all). However, on days 21 and 42, FBS was significantly reduced in all propolis and MSCs treatment groups (p<0.001 for all) (Figure 5).

### Serum albumin concentration

**Figure 6:**
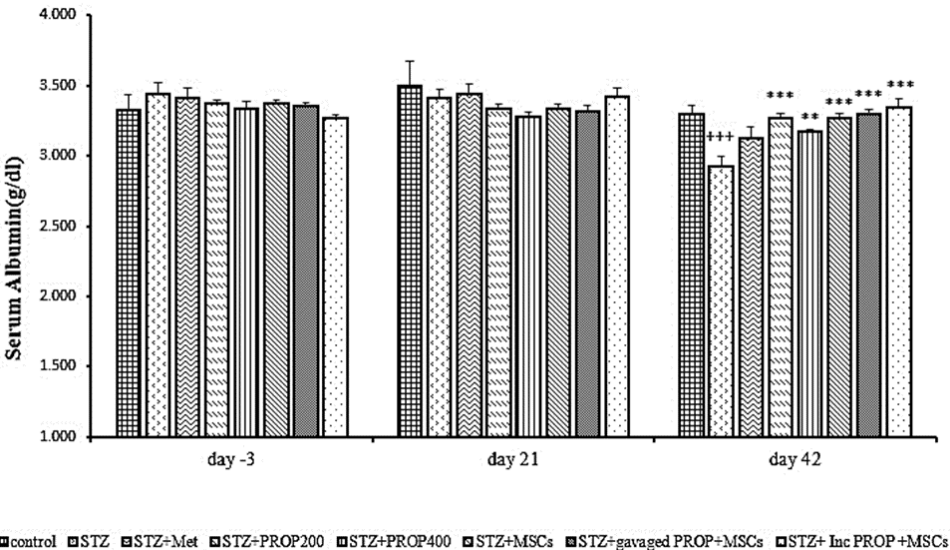
Serum albumin concentration in all experimental groups. Values are mean±SEM (n = 6 per group) and were analyzed using one-way ANOVA followed by Tukey's post hoc test. +++p<0.001 compared to the control group. **p<0.01, ***p<0.001 compared to STZ group. STZ: Streptozotocin; Met: Metformin; PROP: Propolis; MSCs: Mesenchymal stem cell, Inc: Incubated.

Figure 6 shows the serum albumin concentration in different study groups. As shown in the Figure, there was no significant difference in serum albumin concentration between the different groups on days -3 and 21 of the study. However, the serum level of albumin on day 42 showed a significant decrease in the diabetic group compared to the control group (p<0.001). The serum albumin concentration in all diabetic groups treated with propolis and MSCs was significantly higher than the diabetic animals (p<0.01 - p<0.001) (Figure 6).

### Serum creatinine concentration

**Figure 7:**
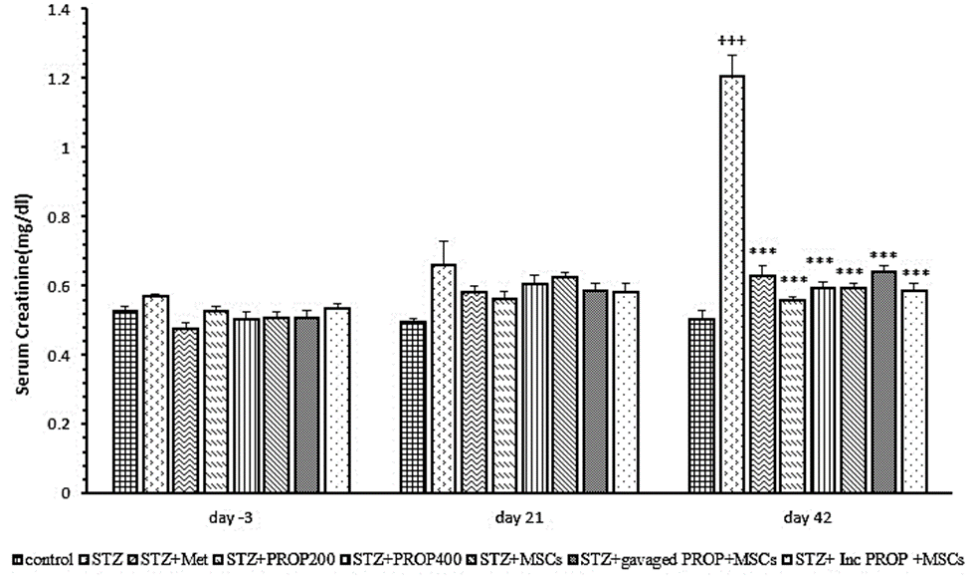
Serum creatinine concentration in all experimental groups. Values are mean±SEM (n = 6 per group) and were analyzed using one-way ANOVA followed by Tukey' post hoc test. +++p<0.001 compared to control group. ***p<0.001 compared to STZ group. STZ: streptozotocin; Met: Metformin; PROP: propolis; MSCs: mesenchymal stem cell, Inc: incubated.

Figure 7 shows the serum creatinine concentration in the different study groups. As shown in the Figure, there was no significant difference in serum creatinine concentration between the different groups on days -3 and 21 of the study. However, serum creatinine concentration on day 42 showed a significant increase in the diabetic group compared to the control group (p<0.001). The serum creatinine concentration in all diabetic groups treated with metformin, propolis and MSCs was significantly lower the diabetic animals (p<0.001 for all) (Figure 7).

### Serum urea concentration

**Figure 8:**
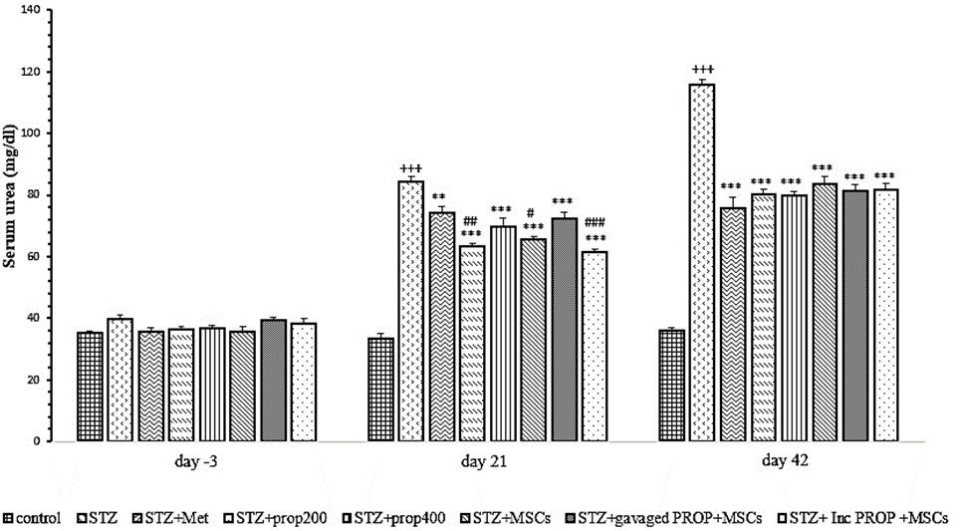
Serum urea concentration in all experimental groups. Values are mean±SEM (n = 6 per group) and were analyzed using one-way ANOVA followed by Tukey's post hoc test. +++p<0.001 compared to control group. **p<0.01, ***p<0.001 compared to STZ group, ^###^p<0.001 compared to STZ+Met group. STZ: Streptozotocin; Met: Metformin; PROP: Propolis; MSCs: Mesenchymal stem cell, Inc: Incubated.

Figure 8 shows the serum urea concentration in the different study groups. As shown in the Figure, serum urea level before STZ injection (Day -3) showed no significant change between different experimental groups. Serum urea concentration on days 21 and 42 showed significant increases in the diabetic group compared to the control group (p<0.001 for both). However, on days 21 and 42 of the study, serum urea concentration was significantly reduced in all diabetic groups treated with metformin, propolis and MSCs when compared to the diabetic group (p<0.01-p<0.001) (Figure 8). Moreover, on day 21, serum urea concentrations of diabetic rats that were treated with propolis (200 mg/kg, p<0.01), MSCs (p<0.05), and MSCs pre-incubated with propolis (p<0.001), were significantly lower than metformin-treated group (Figure 8). 

### Kidney index and histopathology

#### Kidney index

**Figure 9:**
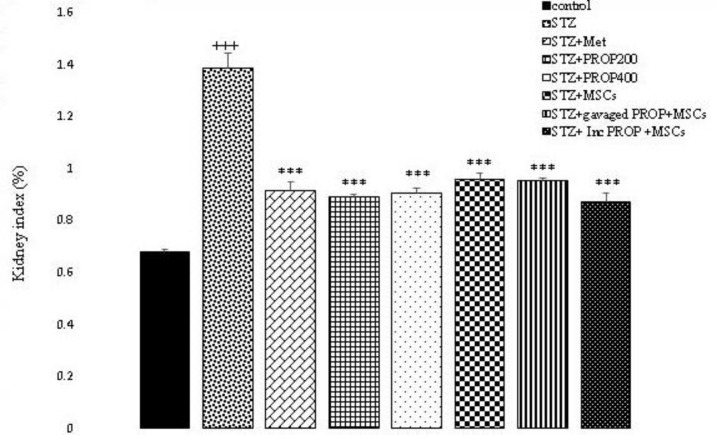
Kidney index in all experimental groups. Values are mean±SEM (n = 6 per group) and were analyzed using one-way ANOVA followed by Tukey's post hoc test. +++ p<0.001 compared to control group, ***p<0.001 compared to STZ group. STZ: Streptozotocin; Met: Metformin; PROP: Propolis; MSCs: Mesenchymal stem cell, Inc: Incubated.

Figure 9 shows the comparison of the kidney index in different experimental groups. As shown in the Figure, the kidney index of the diabetic animals significantly increased compared with the control group (p<0.001). However, in all treated groups, the kidney index significantly reduced compared with the STZ group (p<0.001) (Figure 9).

### Histopathology

**Figure 10:**
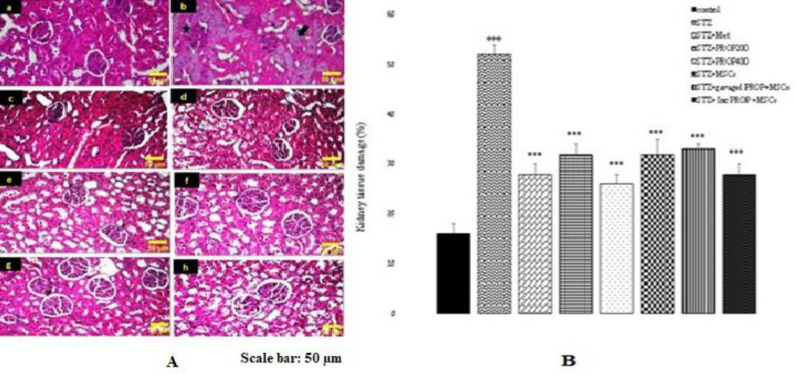
(A) Light microscopy of renal sections in experimental groups. The morphology of the kidneys in control animals was normal. Tubular cell necrosis (double-headed arrow), flattening of the proximal epithelial cells, loss of brush border, tubular lumen dilatation (thin arrow), vacuolated cytoplasm (thick arrow) and severe glomerular changes (star) were all observed in the STZ group. In all treated diabetic rats, the histopathological features improved in the kidney (hematoxylin and eosin, ×200). (B) The values represent mean ± SEM. ^+++^p<0.001 compared to control group. ***p<0.001 compared to STZ group. a: control; b: STZ; c: STZ+Met; d: STZ+PROP200; e: STZ+PROP400; f: STZ+MSCs; g: STZ+gavaged PROP+MSCs; h: STZ+Inc PROP+ MSCs. STZ: Streptozotocin; Met: Metformin; PROP: Propolis; MSCs: Mesenchymal stem cell, Inc: Incubated.

Figure 10 shows the photomicrographs of H&E staining of kidney tissues of the experimental groups. Kidney tissue sections from the control animals showed normal architecture (Figure 10 A and B). However, STZ injection caused tubular cell necrosis, flattening of the proximal epithelial cells, loss of brush border, tubular lumen dilatation, intraluminal casts, vacuolated cytoplasm and severe glomerular changes in the STZ-diabetic group (Figure 10 A and B). In comparison to the STZ group, in all treated diabetic rats, the kidney histopathological features significantly improved compared to the STZ group (p<0.001 for all) (Figure 10 B). 

## Discussion

Urinary albumin excretion, progressive decline in glomerular function and loss of podocytes are among the major pathological and clinical features of diabetic nephropathy (Lewis et al. 1993; Noshahr et al. 2020).

In this study, we induced hyperglycemia by STZ injection, leading to alteration in FBS, serum creatinine, urea and albumin levels, kidney index and kidney histopathological injuries.

Propolis is shown to have various beneficial effects on several chronic diseases like DM (Diabetes mellitus) and renal dysfunction. Antioxidant, anti-inflammatory, antihyperglycemic and antihypertensive actions are among the diverse pharmacologic effects of propolis (Rivera-Yañez et al. 2020).

There are also numerous studies about the ability of MSCs to treat diabetic nephropathy (Ezquer et al. 2009; Ezquer et al. 2008). 

Currently, MSCs are treated with individual and mixtures of crude herbal extracts to determine the mechanisms and effects of herbs on stem cell growth and differentiation (Udalamaththa et al. 2016). MSCs are attractive candidates for renal repair because nephrons have mesenchymal origin and stromal cells play a critical role in signaling, which causes the nephron and collecting ducts to differentiate (Anglani et al. 2004).

Given the lack of research in this area about the co-administration of propolis and stem cells for the treatment of diabetic nephropathy, it appears that the processes of their actions are that to regulate the fate of stem cells, many cytokines and growth factors have been brought in, including those that promote self-renewal and differentiation from the initial niche (Kopp et al. 2005). 

According to the results of the current study, diabetic rats treated with propolis showed a decline in FBS which was in agreement with the result of EL Rabey et al who used propolis in their investigation (El Rabey et al. 2017). It has been reported that propolis exerts its hypoglycemic effect through a positive effect on the phosphorylation of insulin receptors (Nie et al. 2017), increased glucose uptake, translocation of the insulin-sensitive GLUT4 (Glucose Transporter type 4) receptor and suppression of gluconeogenesis genes pathways (Ueda et al. 2013).

In our study, the blood glucose of diabetic rats was suppressed in the group treated with MSCs, which is along with the results of other studies (Ezquer et al. 2008). Additionally, combination therapy or pre-incubation of MSCs with propolis was able to reduce blood glucose in diabetic animals. 

In our results, propolis and MSCs significantly diminished serum creatinine in all treated diabetic groups compared to the STZ-diabetic rats on the 42^nd ^day of the experiment. The present results are in agreement with the findings of similar studies (El Menyiy et al. 2019; Ezquer et al. 2015). 

Moreover, the current study showed that propolis, MSCs and their combination were able to increase serum albumin significantly on the 42^nd^ day of the experiment compared with the STZ-diabetic group. El Menyiy et al. in 2019 also reported that propolis extract increases serum albumin in comparison to the STZ group (El Menyiy et al. 2019).

Additionally, Ezquer et al have reported that BM-MSCs injection to STZ-induced type 1 diabetic mice decreases albuminuria (Ezquer et al. 2009).

Our results also show that propolis and MSCs administration lowers serum urea in all treated animals in comparison to the STZ group. El Menyiy et al.'s findings support this outcome, which demonstrated that propolis considerably reduced serum urea levels in comparison to diabetic rats (El Menyiy et al. 2019). 

In our experiment, the kidney index of all propolis and MSCs treated groups declined in comparison to the STZ-diabetic group. This finding confirms the report of other studies that revealed that propolis extract and MSCs enhanced renal weights (Abo-Salem et al. 2009; Lv et al. 2014).

Our findings also revealed that renal histopathologic alterations including dilation of renal tubules, cast formation, lymphocyte infiltration, cytoplasmic vacuoles, and glomerular degeneration significantly improved in all treatment groups compared with the STZ group. Our results are along with reports that show that MSC and MSC-conditioned medium therapies suppressed pathological changes significantly in the glomerulus, tubular epithelial cells and the interstitium, resulting in the repair of renal tissue in STZ-induced diabetic mice via the paracrine effect of trophic factors including exosomes. These extracellular vesicles are thought to be significant mediators of cell-to-cell communication, facilitating the transfer of proteins, receptors, and genetic information (mRNA and microRNAs) as well as the direct activation of target cells by MSCs (Biancone et al. 2012).

It is probable that propolis extract via activating MMP2 (Matrix Metallopeptidase 2) and MMP9 prevents basement membrane thickening, and mesangial matrix expansion, and protects against glomerular sclerosis and fibrosis injuries in renal tissue of diabetic rats (Shah et al. 2007). Through the manipulation of cytokines, exogenous phytochemicals have the potential to directly or indirectly impact stem cell biology. The rejuvenating properties of herbal medicine are thought to result from their interactions with adult stem cell activity.

Therefore, we raised the hypothesis that propolis and MSCs combination cause synergic effects on various injuries exerted by diabetic nephropathy and probably boost mentioned treatment-related pathways using paracrine effects.

Current results of our experiment indicate that MSCs, propolis, and their combination effectively reduce FBS, serum urea, creatinine levels, renal index, and histopathological damage in diabetic rats compared to the STZ group. Notably, propolis and MSCs, along with their combination, show greater positive effects on blood glucose and albumin levels in diabetic rats than metformin, a widely recognized antidiabetic medication. Additionally, the duration of treatment plays a crucial role in maximizing the beneficial effects of propolis, MSCs, and their combination in diabetic subjects. Nevertheless, further research is necessary to elucidate the precise mechanisms by which propolis, MSCs, and their combination improve DKD.
